# A Rare Case of Acute Vasitis

**DOI:** 10.7759/cureus.51337

**Published:** 2023-12-30

**Authors:** Saleh Al-Gburi, Snehal Patel

**Affiliations:** 1 Urology, Wirral University Teaching Hospitals, Wirral, GBR

**Keywords:** vas deferens, infection, urology surgery, right inguinal hernia, vasitis

## Abstract

Inflammation of the vas deferens, or vasitis, is a rarely reported condition that can manifest as either acutely painful infectious vasitis or predominantly asymptomatic vasitis nodosa. Acute vasitis is usually presented with ambiguous clinical findings, and a scan is required for a definitive diagnosis. Retrograde urinary pathogens are typically the cause, and it is treatable conservatively. We present a male in his 40s with a one-day history of right groin pain and a history of right indirect inguinal hernia. On examination, there was an impression of an incarcerated inguinal hernia. A CT scan revealed thickening and inflammatory changes associated with the inguinal canal and a picture of the rare inflammatory condition, acute vasitis. This case report illustrates the significance of understanding the wide range of possible diagnoses associated with acute groin pain and swelling and the importance of imaging in the diagnosis, which might help avoid needless operation.

## Introduction

Vasitis, or inflammation of the vas deferens, is a seldom reported illness that can be acutely painful infectious vasitis or largely asymptomatic vasitis nodosa [1]. Clinically, it manifests as nonspecific inguinal swelling and local discomfort that can be misdiagnosed as orchitis, epididymitis, testicular torsion, or inguinal hernia. Being familiar with symptoms, imaging findings, and differential diagnosis is essential. It is crucial to avoid unneeded surgery, particularly when symptoms resemble an inguinal hernia [2]. Escherichia coli and other common urinary pathogens, as well as Chlamydia trachomatis and Mycobacterium tuberculosis, are the main causes [2,3].

We present a rare case of acute vasitis to illustrate the significance of understanding the wide range of possible diagnoses associated with acute groin pain and swelling and the importance of imaging in the diagnosis of this condition, which can be treated conservatively with an antibiotic course.

## Case presentation

A male in his 40s presented to the accident and emergency department with a one-day history of right groin pain. The patient didn't complain of a fever or vomiting. He had no bowel symptoms, and the bowels were normally opened on the presentation day. He has a history of right-indirect inguinal hernia and epilepsy. However, he has no history of urinary tract infection, recent trauma, or previous surgery. On examination, there was a right excruciatingly tender reducible inguinal hernia and a soft, non-tender abdomen. A testicular examination reveals a normal position and non-tender testis without swelling. An impression of an incarcerated hernia was made.

**Figure 1:**
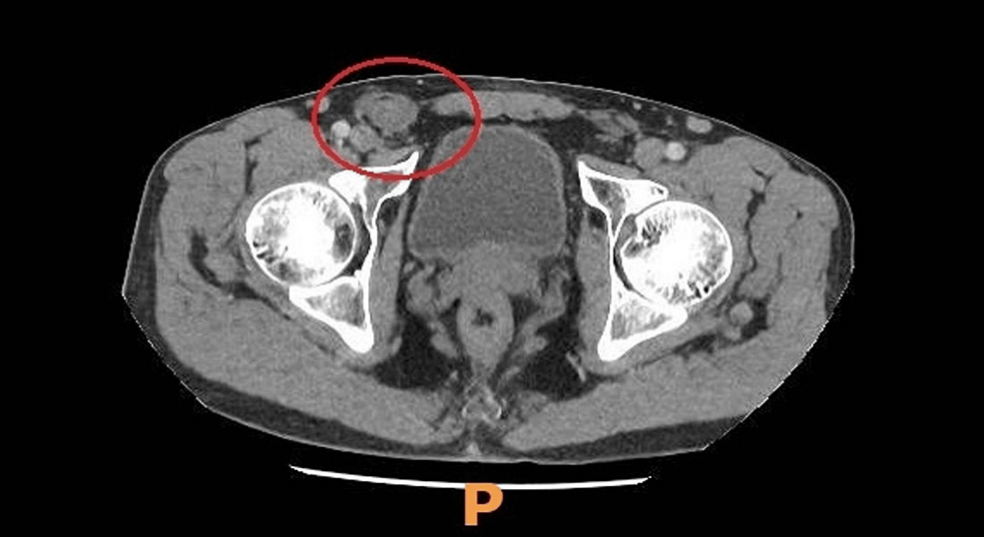
An axial view of a CT scan of the abdomen and pelvis with contrast The scan shows thickening and inflammatory changes associated with the inguinal canal likely secondary to inflammation of the contained sperma.

**Figure 2:**
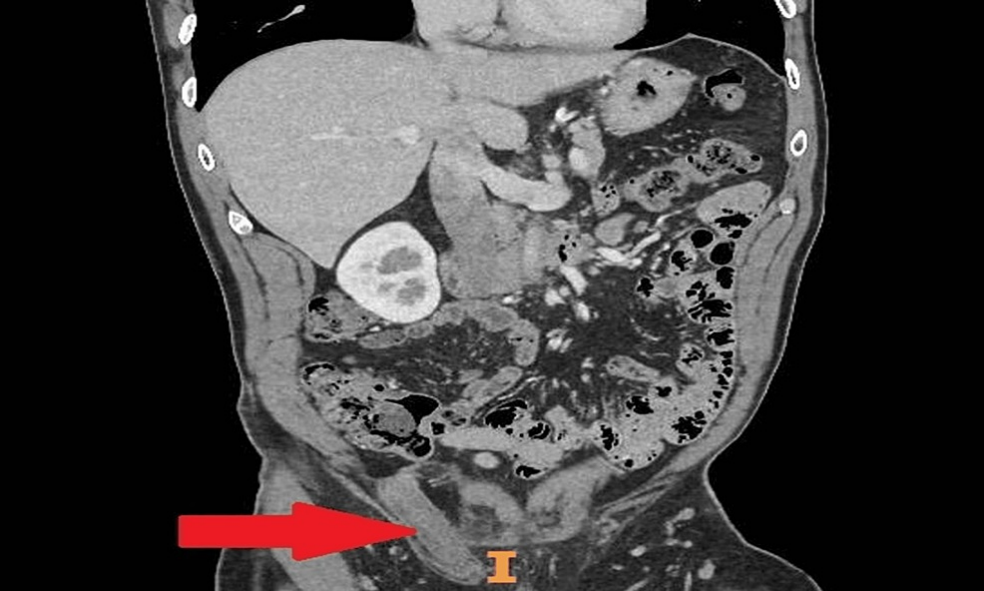
A coronal view of a CT scan of the abdomen and pelvis with contrast The scan shows thickening and inflammatory changes associated with the inguinal canal likely secondary to inflammation of the contained sperm.

He had an elevated lactate level of 2.9 mmol/L, and the inflammatory markers were normal with normal kidney function tests and liver function tests. His urinalysis was normal, and his urine culture was negative. A CT of the abdomen and pelvis with contrast revealed normal caliber small and large bowel loops throughout, with no evidence of obstruction and no bowel-containing hernia identified. In the right groin, there were thickening and inflammatory changes associated with the inguinal canal, likely secondary to inflammation of the contained spermatic cord. CT findings are illustrated in Figures 1-2. Combining the history, clinical symptoms, and imaging helped to make the diagnosis of vasitis.

The patient was admitted to the ward for one night and treated with trimethoprim and analgesia. He was discharged the next day with trimethoprim 200 mg twice daily for 14 days, as per hospital protocol. He returned to his daily activities and his work. At follow-up three weeks later, his pain significantly improved.

## Discussion

Vasitis is an inflammatory condition that includes vasitis nodosa and acute vasitis. Acute vasitis is uncommon and often manifests as an immediate, painful inguinal lump, which may be accompanied by leucocytosis and fever [1]. The retrograde spread of common urinary pathogens such as E. coli is assumed as the etiology of acute infectious vasitis. Urine culture, on the other hand, is frequently negative. Other uncommon pathogens, Chlamydia trachomatis and Mycobacterium tuberculosis, have both been reported [2,3].

The diagnosis of vasitis is confused by the disease's rarity as well as the ambiguous clinical findings. CT findings include vas deferens thickening, spermatic cord edema, and peripheral adipose stranding. CT or MRI should be applied to more precisely delineate the anatomy and rule out an incarcerated inguinal hernia, as supported by the evidence [4,5].

According to the current literature, the majority of reported vasitis can be treated with anti-inflammatories and antibiotics alone [4,6,7]. A case of pediatric vasitis was documented wherein inguinal enlargement symptoms experienced only a partial remission following a seven-day course of oral antibiotic treatment. Subsequently, the child was admitted to the hospital and successfully managed with intravenous antibiotics [8].

The more frequent vasitis nodosa manifests as an asymptomatic inguinal mass and is encountered in individuals who have had vas deferens manipulation, such as vasectomy, prostatectomy, or non-mesh herniorrhaphy [9]. Vasitis nodosa, first reported by Benjamin in 1943, is a benign chronic inflammation that results in fusiform nodular thickening of the vas deferens [10].

On review of the literature to date, there have been only nine reported cases (see Table 1). We would like to highlight that this is probably more common than the literature suggests. The age range is between 27 and 55 years old, but it can occur in children and adolescents. The usual presentation is pain in the groin or testis, with or without groin swelling, and is associated with elevated inflammatory markers. Most cases needed a CT scan for a definitive diagnosis and were treated with a course of antibiotics.

**Table 1:** A literature review of recent publications of acute vasitis case reports

References	Patient medical and surgical history	Symptoms	Signs	Laboratory	Scans	Management
Chen et al., 2019 [2]	Middle-aged patient, normally fit and well	Severe right inguinal and lower abdominal pain.	Tenderness with swelling in the right inguinal area.	Leukocytosis and elevated C-reactive protein (CRP), negative urine culture.	CT scan: the right spermatic cord and vas deferens exhibit diffuse edematous alterations.	500 mg of levofloxacin once daily for two weeks
Marcos et al., 2017 [11]	42-year-old patient on hormonal therapy after bilateral orchiectomy at age 13 because of cryptorchidism	A three-day history of abdominal pain, mainly on the right iliac fossa and hypogastrium. He had a history of 10 days of urethral discharge, which was resolved before the pain developed.	Epigastric and right iliac fossa (RIF) tenderness, a small, painful, irreducible hernia in the right groin.	Leukocytosis and elevated C-reactive protein.	US scan: an inguinal hernia was observed, which covered a peristaltic tubular. Structure originating from the abdominal cavity. This structure exhibited pain upon compression. CT scan: the tip of an inflamed appendix was encased within a right inguinal hernia.	One-port laparoscopy showed no inflamed appendix and inflammation of the vas deferens.
Schurr et al., 2014 [8]	Six-year-old patient	Pain and swelling in the left groin, with a history of pain and swelling in the left testicles.	Oedema and tenderness of the left groin, with consistent tenderness in the left testis.		US scan: enlarged left epididymis with increased vascular flow, left groin lymphadenopathy. CT scan: confirmed an inflamed spermatic cord and excluded inguinal hernia.	IV antibiotic and seven days of oral antibiotics
Lin et al., 2019 [12]	39-year-old patient	Right groin and scrotum pain for two days.	A palpable and tender inguinal mass.	Leukocytosis and elevated C-reactive protein.	CT scan: the right spermatic cord and vas deference exhibited a noticeable thickening, accompanied by edematous alterations and peripheral fat stranding.	Oral ciprofloxacin antibiotics
Eddy et al., 2011 [5]	40-year-old patient	Left groin pain radiated to the scrotum for 36 hours.	Inguinal mass		Duplex Doppler: showed normal blood flow in the left testicles. CT scan: edematous spermatic cord with effacement of fat planes around the vas deferens.	Course of antibiotics
Bomar et al., 2020 [13]	14-year-old patient who had surgically corrected left inguinal hernia and ipsilateral renal agenesis	Left testicular pain and swelling.	Swollen and edematous left hemiscrotum and soft, tender inguinal mass.		US Doppler: normal blood flow to the testis, mildly hypervascular epididymal. CT scan: supratesticular lesion and left spermatic cord thickening.	IV 1 gm of ceftriaxone + sulfamethoxazole-trimethoprim 800-160 mg PO BID for seven days
Almutairi et al., 2022 [14]	27-year-old patient, normally fit and well	Severe left scrotal pain and swelling for four days, one instance of gross hematuria, and inguinoscrotal swelling were additionally observed.	The left hemiscrotum was extremely tender and swollen, with a tender mass over the left inguinal region.	Leukocytosis	US of the testis: the left testis and epididymis exhibited marginally increased vascularity. CT scan: left epididymal head hyperenhancement accompanied by vascular engorgement, swelling, and substantial thickening of the left spermatic cord.	Single dose ceftriaxone IM and levofloxacin
Jones et al. 2020 [15]	52-year-old patient with a history of vasectomy, then reverse vasectomy, and then re-do vasectomy, with a history of complex regional pain syndrome affecting his left hand and right foot	Left testicular pain.	Exquisitely tender left vas deferens.	Normal inflammatory markers.	US scan: reported left vasitis.	Oral ciprofloxacin
Patel et al. 2014 [4]	55-year-old patient, normally fit and well	Severe left groin mass associated with mass.	A mass that is indurated, hard, and distended in the left inguinal region.	Leukocytosis and elevated C-reactive protein.	US scan: left spermatic cord that was diffusely swollen along its entire length. MRI: extensive, diffuse edema tracking along the spermatic cord.	Intravenous gentamicin, clindamycin, and oral ciprofloxacin

## Conclusions

Acute vasitis is an inflammatory condition caused by a retrograde infection of common urinary pathogens. It usually presents with pain or swelling in the groin and can occur in every age group. The diagnosis might be challenging because of its rarity, but it should be on the differential diagnosis list. An imaging CT scan or MRI scan will prevent inappropriate surgery in such patients with a history of inguinal hernia, and the diagnosis of acute vasitis can be achieved after a combination of history, clinical examination, and imaging. It can be treated with an antibiotic course, according to local hospital policy.
